# Modification of CeNi_0.9_Zr_0.1_O_3_ Perovskite Catalyst by Partially Substituting Yttrium with Zirconia in Dry Reforming of Methane

**DOI:** 10.3390/ma15103564

**Published:** 2022-05-16

**Authors:** Mahmud S. Lanre, Ahmed E. Abasaeed, Anis H. Fakeeha, Ahmed A. Ibrahim, Abdullah A. Alquraini, Salwa B. AlReshaidan, Ahmed S. Al-Fatesh

**Affiliations:** 1 Chemical Engineering Department, College of Engineering, King Saud University, P.O. Box 800, Riyadh 11421, Saudi Arabia; mahmudsofiu@gmail.com (M.S.L.); anishf@ksu.edu.sa (A.H.F.); abalquraini@ksu.edu.sa (A.A.A.); 2 Department of Chemistry, College of Science, King Saud University, Riyadh 11451, Saudi Arabia; chem@ksu.edu.sa

**Keywords:** catalyst stability, dry reforming of methane, perovskite catalyst, catalyst activity

## Abstract

Methane Dry Reforming is one of the means of producing syngas. CeNi_0.9_Zr_0.1_O_3_ catalyst and its modification with yttrium were investigated for CO_2_ reforming of methane. The experiment was performed at 800 °C to examine the effect of yttrium loading on catalyst activity, stability, and H_2_/CO ratio. The catalyst activity increased with an increase in yttrium loading with CeNi_0.9_Zr_0.01_Y_0.09_O_3_ catalyst demonstrating the best activity with CH_4_ conversion >85% and CO_2_ conversion >90% while the stability increased with increases in zirconium loading. The specific surface area of samples ranged from 1–9 m^2^/g with a pore size of 12–29 nm. The samples all showed type IV isotherms. The XRD peaks confirmed the formation of a monoclinic phase of zirconium and the well-crystallized structure of the perovskite catalyst. The Temperature Program Reduction analysis (TPR) showed a peak at low-temperature region for the yttrium doped catalyst while the un-modified perovskite catalyst (CeNi_0.9_Zr_0.1_O_3_) showed a slight shift to a moderate temperature region in the TPR profile. The Thermogravimetric analysis (TGA) curve showed a weight loss step in the range of 500–700 °C, with CeNi_0.9_Zr_0.1_O_3_ having the least carbon with a weight loss of 20%.

## 1. Introduction

Fossil fuels such as natural gas, coal, and crude oil serve as the backbone for the 21st century energy, industrial, and transportation sectors of the economy [1]. Their usage liberates CO_2_ that accumulates over time in the atmosphere depleting the ozone layer shield of the earth, leading to the rising temperature of the earth causing global warming. The impact of this global warming on the environment includes ecosystem collapse, desertification, etc., and can have direct effects on humans such as extremely hot weather conditions, or indirect effects such as crop failure and farmland loss causing a food shortage. In order to save the earth, the emissions of the greenhouse gases must be drastically reduced; hence, this necessitates dry reforming of methane (DRM) reaction which employs methane and carbon dioxide greenhouse gases to produce syngas (H_2_/CO), thereby controlling the mitigation of these two global warming gases [2]. DRM can also utilize biogas, composed mainly of CO_2_ and CH_4_. This reaction is strongly endothermic.

The dry reforming reaction comprises:

Methane Dry Reforming: CO_2_ + CH_4_ ↔ 2CO + 2H_2_ (1)

Reverse Water-Gas Shift Reaction: CO_2_ + H_2_ ↔ CO + H_2_O (2)

Methane Cracking Reaction: CH_4_ ↔ C + H_2_ (3)

Disproportionation Reaction: 2CO ↔ C + CO_2_ (4)

Carbon Gasification: C + H_2_O ↔ CO + H_2_ (5)

Synthesis gas (syngas) obtained from DRM reaction can be converted to methanol and synthetic fuels by Shell middle distillate synthesis (SMDS) or Fischer–Tropsch syntheses [3]. Hydrogen production is being increased during the conversion of CO with steam in water gas shift reaction to CO_2_ [1]. Suitable catalysts for the DRM have been investigated using transition elements like Ni, Co, Pd, Ir, and perovskite-type oxides, which are very active [4]. Nickel catalysts are majorly considered for dry reforming reactions [5,6,7,8] based on its low price. However, coking and sintering cause the catalyst to deactivate [9,10]. The two major characteristics of the catalyst that defines coking are surface properties and acidity [11]. Carbon deposition depends on factors such as the nature of the hydrocarbon and the catalyst and reaction operating conditions [11]. Coking can be mitigated by supporting the active metal on a metal oxide with strong basicity [11]. Methane adsorption on nickel requires the bond breaking of C-H. The CH_4_ is converted thus: CH_4_→CH_3_*→CH_2_*→CH*→C* [11]. Ni catalysts are being bolstered so as to hamper carbon deposits and achieve good stability leading to the development of catalysts with an abundance of oxygen for coke gasification such as perovskite catalyst [12].

The structural stability of perovskite is altered by incorporating an atom into its structure resulting in changes in oxygen mobility [13]. It was reported that lower valent cations addition into perovskite catalyst structure resulted in oxygen ions adsorption onto the surfaces and changes to lattice oxygen. Such changes to the surface lattice oxygen ions would lead to various types of adsorption onto the surface of the perovskite catalyst structure and different mass transfer rates of oxygen [14]. Investigating the influence of cerium on nickel-based catalysts for hydrogen production and reported that the addition of the right amount of cerium can increase oxygen vacancies formation, which can activate oxygen-containing compounds to react with carbon species as soon as it forms [15]. Basically, zirconia is known to be a better support for active metal, due to heat stability and explicit characteristics such as acid-base and reduction-oxidation properties [16,17,18,19,20]. It has been proven that ZrO_2_ is a proper support for Ni, as it gives restricted coke formation with a small carbon combustion temperature [21]. Yttrium oxide (Y_2_O_3_) can either be acting as a promoter or support as a result of its unique chemical and thermal properties. Y_2_O_3_ supported Ni catalyst allows the ease reduction, better activity, stability, and limits the reverse of water gas shift (RWGS) reaction [22]. Yttria-zirconia as support for DRM suggests that the supported Ni catalysts will gain from redox properties of the material which is exceptional to limit the carbonaceous deposit formation thereby increasing the lifetime of the catalyst [23,24,25]. Promoters like alkali earth and alkali with rare earth metals have been used for the activity and stability enhancement of nickel-based catalysts [26,27,28]. Doping of trace amounts of noble metals to Ni catalyst leads to direct improvements of the catalytic reaction features of DRM [29,30,31].

The aim of this work is to study the effect of CeNi_0.9_Zr_0.1_O_3_ perovskite catalyst modified with yttrium on DRM. The incorporation of yttrium into the perovskite structure is evaluated in terms of catalyst activity, stability, amount, and nature of carbon formed.

## 2. Materials and Methods

The perovskite catalysts CeNi_0.9_Zr_0.1−*x*_Y_*x*_O_3_ (*x* = 0, 0.03, 0.05, 0.07, and 0.09) were prepared by the sol-gel method with propionic acid acting as a solvent, to dissolve nitrates of each metal. In the preparation, Ni (NO_3_)_2_·6H_2_O (Sigma, St. Louis, MO, USA), Y(NO_3_)_3_·6H_2_O (Sigma), Ce (NO_3_)_3_·6H_2_O (Sigma), Zr (NO_3_)_4_·6H_2_O (Sigma), and propionic acid (C_3_H_6_O_2_, Sigma) were used. The nitrates were separately dissolved in propionic acid, stirred, and heated at T = 90 °C with oil as a heating medium. Afterward, the solutions were continuously stirred for about 2 h at T = 130 °C. Thereafter, the propionic acid was evaporated with a rotary evaporator at T = 70–80 °C until a gel was formed. The gel obtained was dried at T = 90 °C overnight, and calcined at 725 °C for 4 h. The catalysts formed after calcination were ground into powder and used for the DRM reaction.

### 2.1. Catalytic Testing

The catalysts were tested for DRM at 800 °C reaction temperature under atmospheric pressure. A packed bed reactor stainless steel reactor (0.0091 m internal diameter; 0.3 m height) was used to perform the experiment. An amount of 0.10 g of catalyst was placed in the reactor on top of glass wool. Stainless steel, sheathed thermocouple K-type, axially positioned close to the catalyst bed was used to determine the temperature during the reaction. Preceding the reaction, activation of the perovskite catalysts was done at 700 °C with H_2_. This lasted for 60 min and the remnant H_2_ was purged with N_2_. During the dry reforming reaction, the feed volume ratio was kept at 3:3:1 for CH_4_, CO_2_, and N_2_ gases, respectively, with a space velocity of 42 L/h./g_cat_. The outlet gas from the reactor was connected to an online Gas Chromatography (GC) with a thermal conductivity detector to analyze its composition. The CH_4_, CO_2_ conversion, and H_2_/CO (syngas ratio) were calculated using Equations (4)–(6):

(6) $$\mathrm{Methane}\;\mathrm{conversion}\;\left(\%\right)=\frac{{\mathrm{CH}}_{4,\mathrm{in}}-{\mathrm{CH}}_{4,\mathrm{out}}}{{\mathrm{CH}}_{4,\mathrm{in}}}*100$$

(7) $$\mathrm{Carbon}\;\mathrm{dioxide}\;\mathrm{conversion}\;=\frac{{\mathrm{CO}}_{2,\mathrm{in}}-{\mathrm{CO}}_{2,\mathrm{out}}}{{\mathrm{CO}}_{2,\mathrm{in}}}*100$$

(8) $$\mathrm{Syngas}\;\mathrm{Ratio}=\frac{{\mathrm{mole}\;\mathrm{of}\;H}_{2}\;\mathrm{produced}}{\mathrm{mole}\;\mathrm{of}\;\mathrm{CO}\;\mathrm{produced}}$$

### 2.2. Catalyst Physicochemical Properties Determination

#### 2.2.1. Nitrogen Physisorption

The perovskite catalysts surface area, as well the pore size distribution, was measured by N_2_ adsorption–desorption at −196 °C using a Micromeritics Tristar II 3020 for porosity and surface area analyzer.

#### 2.2.2. Hydrogen Temperature Programmed Reduction Analysis

A total of 70 mg of the sample was loaded inside the TPR sample holder of a Micromeritics apparatus. Thereafter, TPR measurements were performed at 150 °C using Ar gas for 30 min and then cooled to ambient temperature. Thereafter, the sample was heated in a furnace up to 800 °C ramping at 10 °C min^−1^, in the atmosphere of H_2_/Ar mixture (1:9 vol. %) at 40 mL/min. The thermal conductivity detector recorded H_2_ consumption during the operation.

#### 2.2.3. Thermo-Gravimetric (TGA) Analysis

Quantification of carbon deposits on the used catalysts was determined by TGA analysis. 10–15 mg of the used catalysts was filled in a platinum pan. Heating was performed at ambient temperature up to 1000 °C at 20 °C min^−1^ temperature ramp. Loss in mass was constantly monitored as the heating progressed.

#### 2.2.4. X-ray Diffraction (XRD) Analysis

The X-ray Diffraction patterns of the perovskite catalysts were recorded on a Miniflex Rigaku diffractometer that was equipped with Cu Kα X-ray radiation. The device was run at 40 mA and 40 kV.

#### 2.2.5. Transmission Electron Microscopy (TEM)

Transmission electron microscopy (JEOL JEM-2100F) with high resolution to give larger magnification was used to carry out the TEM measurement of both the fresh and used catalyst. The electron microscope operated at 200 kV produces the active metal nickel particle sizes and depicts the morphology of carbon deposit on the used catalyst. Before the TEM measurement, the catalysts were first dispersed ultrasonically in ethanol at room temperature. Thereafter, the drop from the suspension was placed in a lacey carbon-coated Copper grid to produce the images.

#### 2.2.6. Laser Raman (NMR-4500) Spectrometer

Laser Raman (NMR-4500) Spectrometer (JASCO, Tokyo, Japan) was used to record Raman spectra of the spent catalyst samples. The wavelength of the excitation beam was set to 532 nm, and an objective lens of 100× magnification was used for the measurement. The laser intensity was adjusted to 1.6 mW. Each spectrum was received by averaging 3 exposures on 10 s. Spectra were recorded in the range 1200–3000 cm^−1^ (Raman shift) and were processed by using Spectra Manager Ver.2 software (JASCO, Tokyo, Japan).

## 3. Results

### 3.1. BET (Brunauer–Emmett–Teller) Analysis

**Figure 1:**
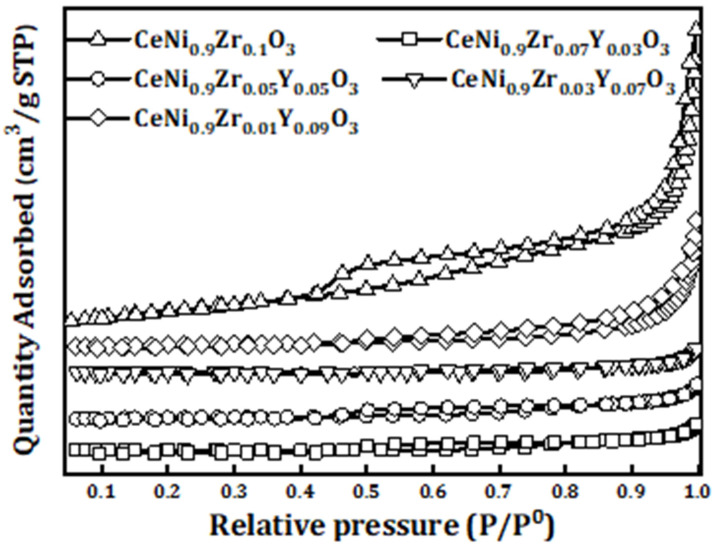
Nitrogen physisorption isotherms of perovskite catalyst CeNi_0.9_Zr_0.1−*x*_Y_*x*_O_3_ (*x* = 0, 0.03, 0.05, 0.07, and 0.09).

The surface area of the catalysts does not vary largely with one another with pore volume less than one. The isotherm curves as shown in Figure 1 suggest that the materials are mesoporous in nature with pore diameter less than 50 nm. The isotherm linear plot of all the samples represents Type IV isotherm. This occurs due to capillary condensation of gases in the tiny pores of solid at pressures below the gas saturation pressure. The textural properties of the fresh catalyst are provided in Table 1.

### 3.2. Temperature-Programmed Reduction (TPR)

**Figure 2:**
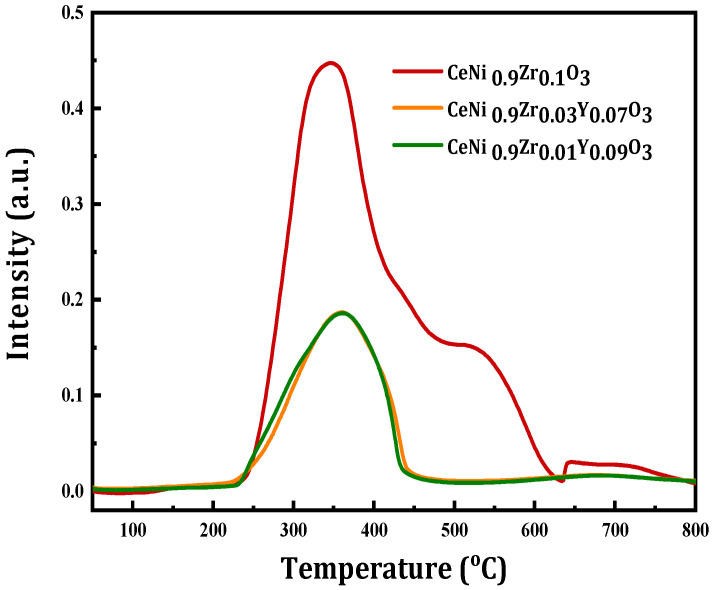
H_2_-TPR profiles of perovskite catalyst CeNi_0.9_Zr_0.1−*x*_Y_*x*_O_3_ (*x* = 0, 0.07 and 0.09).

The active sites for the catalyst samples are Ni and Zr, the samples are activated by H_2_ reduction precedent to reaction. The reducibility of the catalyst samples was performed as shown in Figure 2. Peaks at the lower region are almost the same but there is a shoulder peak for un-modified sample in the moderate temperature region in the TPR profile. It has been reported that the reduction becomes easier while the energy of the metal–oxygen bond decreases [32]. The peaks at moderate temperature regions can be attributed to the reduction of Ni^3+^ to Ni^2+^.

### 3.3. X-ray Diffraction (XRD) Analysis

**Figure 3:**
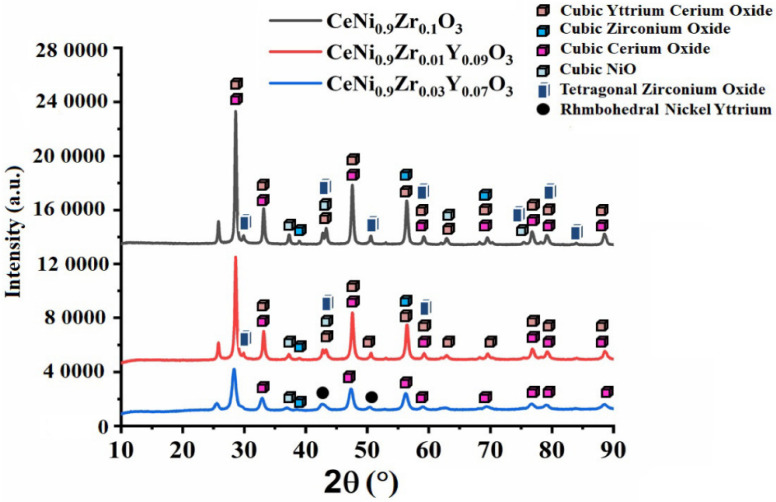
XRD pattern of perovskite catalyst CeNi_0.9_Zr_0.1−*x*_Y_*x*_O_3_ (*x* = 0.00, 0.07 and 0.09).

**Figure 4:**
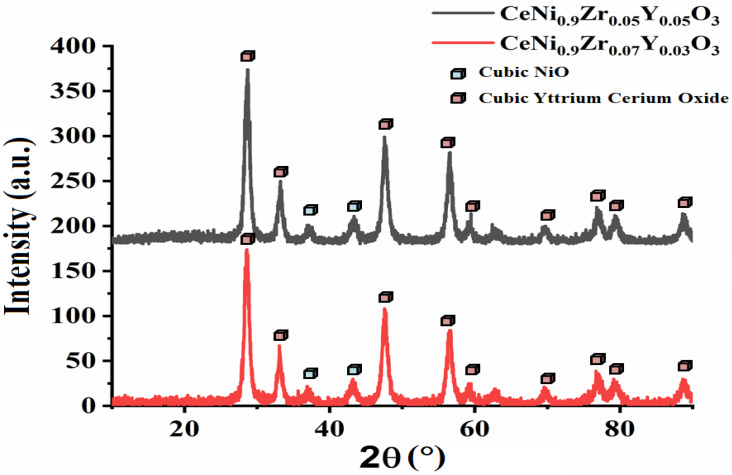
XRD pattern of perovskite catalyst CeNi_0.9_Zr_0.1−*x*_Y_*x*_O_3_ (*x* = 0.03 and 0.05).

The XRD peaks of the perovskite catalysts are depicted in Figure 3 and Figure 4. At Y = 0.03–0.05, no XRD peaks of ZrO_2_ phases are observed. In other samples, cubic zirconia oxide (JCPDS card reference number 00-020-0684) or tetragonal zirconium oxide (JCPDS card reference number 01-081-1547) or both phases are found. The XRD peaks confirm the formation of tetragonal and cubic zirconia [33,34]. The peaks from the XRD include cubic CeO_2_ (JCPDS card reference number 01-002-1306), cubic NiO (JCPDS card reference number 00-002-1216), and cubic yttrium cerium oxide (JCPDS card reference number 01-075-0177). For the CeNi_0.9_Zr_0.1_O_3_ catalyst_,_ the intensity of the peaks at 2θ = 28.5° and 48.2° are higher, suggesting a discrete crystalline phase of CeO_2_ formation. The Ni in the Ce-Ni systems exists as NiO on the ceria surface and Ni^2+^ ions in the CeO_2_ lattice [35,36]. The catalyst CeNi_0.9_Zr_0.03_Y_0.07_O_3_ had an additional rhombohedral nickel yttrium phase (JCPDS card reference number 00-020-0646).

### 3.4. Transmission Electron Microscope (TEM) Analysis

**Figure 5:**
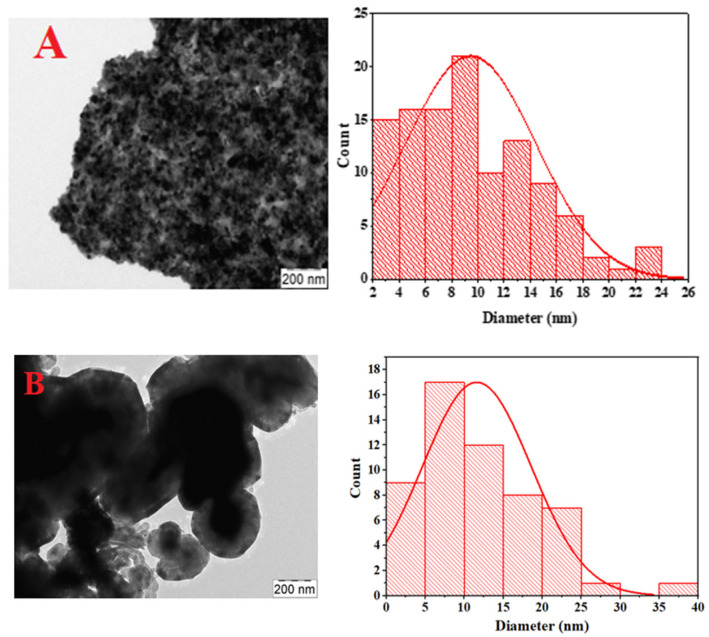
TEM micrographs and matching particle size distribution for fresh CeNi_0.9_Zr_0.1_O_3._ (**A**) and used CeNi_0.9_Zr_0.1_O_3._ (**B**) catalyst.

**Figure 6:**
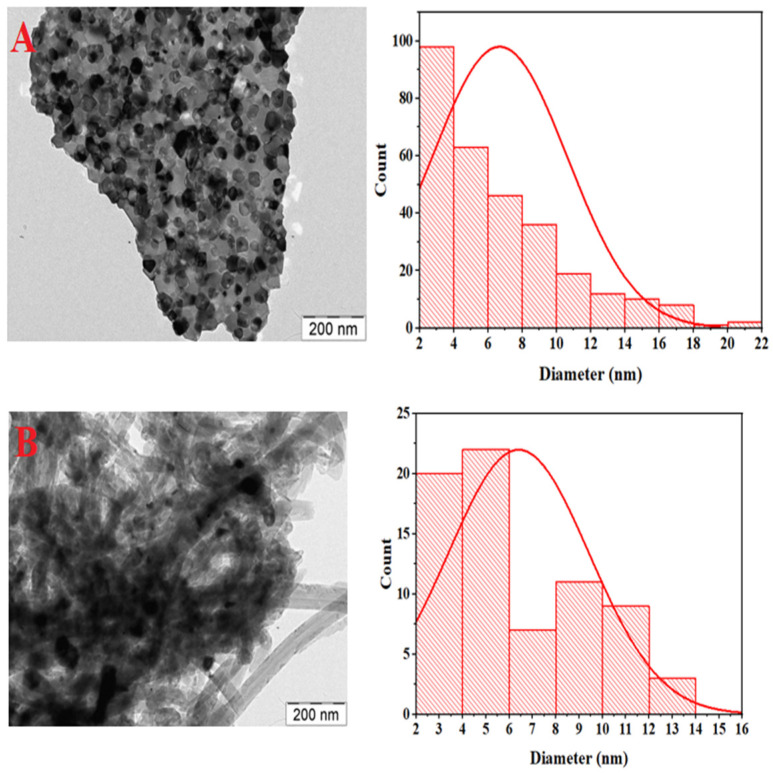
TEM micrographs and matching particle size distribution for fresh CeNi_0.9_Zr_0.01_Y_0.09_O_3._ (**A**) and used CeNi_0.9_Zr_0.01_Y_0.09_O_3._ (**B**) catalyst.

The TEM analysis for both the fresh and spent catalysts are illustrated in Figure 5 and Figure 6 at 200 nm magnification. The used CeNi_0.9_Zr_0.01_Y_0.09_O_3_ catalyst particles are shown with the carbon spreading across the surface. The nickel particle size distribution expressed in the nanometer is plotted for each of the TEM images with that of the spent catalyst higher than the fresh catalyst samples and the result tabulated in Table 2.

### 3.5. Catalyst Activity

**Figure 7:**
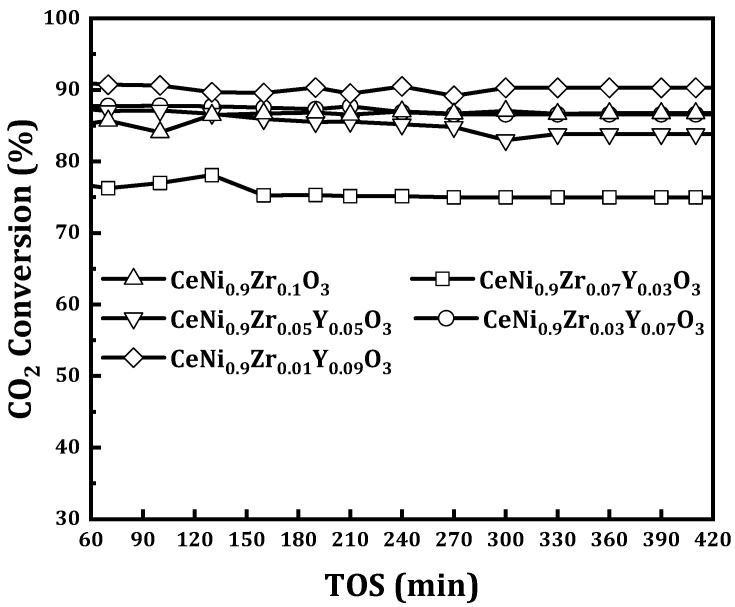
CO_2_ conversion of perovskite catalysts CeNi_0.9_Zr_0.1−*x*_Y_*x*_O_3_ (*x* = 0, 0.03, 0.05, 0.07, and 0.09) at 800 °C, 1 atmosphere and GHSV = 42 L/(h.g_cat_).

**Figure 8:**
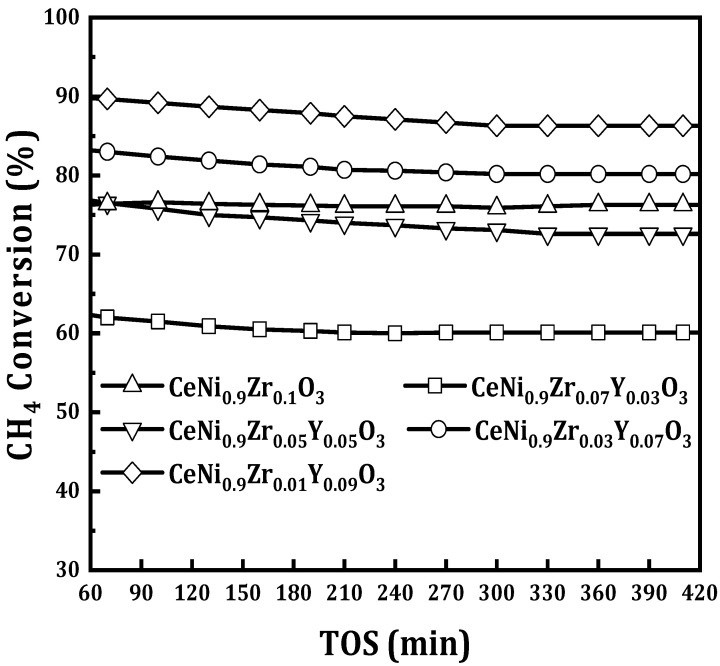
CH_4_ conversion of perovskite catalysts CeNi_0.9_Zr_0.1−*x*_Y_*x*_O_3_ (*x* = 0, 0.03, 0.05, 0.07, and 0.09) at 800 °C, 1 atmosphere and GHSV = 42 L/(h.g_cat_).

**Figure 9:**
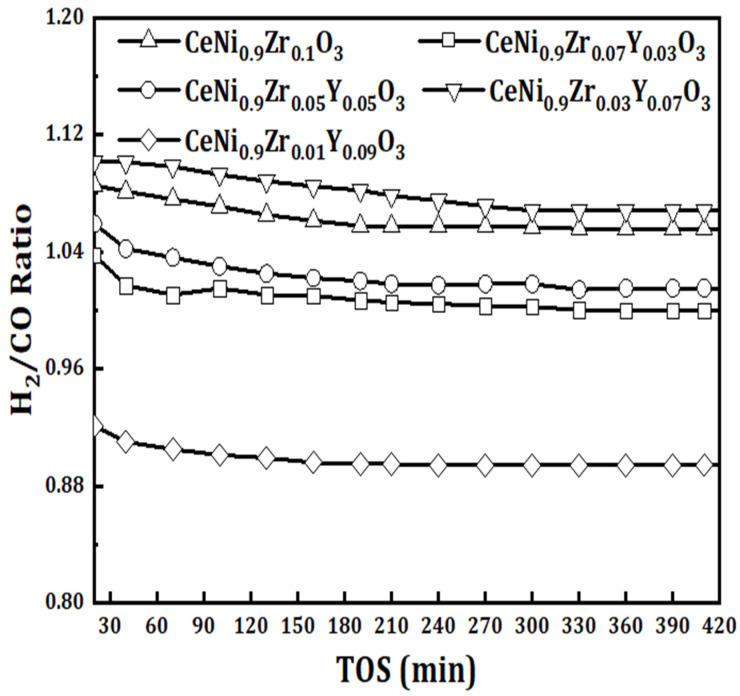
H_2_/CO ratio of perovskite catalysts CeNi_0.9_Zr_0.1−*x*_Y_*x*_O_3_ (*x* = 0, 0.03, 0.05, 0.07, and 0.09) at 800 °C, 1 atmosphere and GHSV = 42 L/(h.g_cat_).

A varying amount of yttrium was used to modify the catalyst CeNi_0.9_Zr_0.1−*x*_Y_*x*_O_3_ with *x* = 0, 0.03, 0.05, 0.07, and 0.09) and tested at 800 °C for DRM reaction. The activity of the yttrium modified catalyst CeNi_0.9_Zr_0.07_Y_0.03_O_3_ was lower than the un-modified catalyst CeNi_0.9_Zr_0.1_O_3_ and had the lowest activity for all the tested catalysts. The activity of the CeNi_0.9_Zr_0.05_Y_0.05_O_3_ catalyst is slightly higher than the un-modified CeNi_0.9_Zr_0.1_O_3_ catalyst, with the latter having 87% CO_2_ and 78% CH_4_ conversion, as shown in Figure 7 and Figure 8. The activity of yttrium modified catalyst CeNi_0.9_Zr_0.1−*x*_Y_*x*_O_3_ when *x* = 0.07 and 0.09 is higher than the un-modified CeNi_0.9_Zr_0.1_O_3_ catalyst. The former has 87% CH_4_ and 90% CO_2_ for *x* = 0.07, and 90% CH_4_ and 91% CO_2_ for *x* = 0.09. Yttrium results in oxygen vacancies formations that are believed to contribute to improved catalytic activity [37,38]. The catalyst activity increases with Y loading increase. Figure 9 displays the syngas ratios. The H_2_/CO ratio values for CeNi_0.9_Zr_0.1_O_3_ and CeNi_0.9_Zr_0.07_Y_0.03_O_3_ catalyst are less than one which suggests that the predominant side reaction is RWGS while CeNi_0.9_Zr_0.05_Y_0.05_O_3_, CeNi_0.9_Zr_0.03_Y_0.07_O_3_, CeNi_0.9_Zr_0.01_Y_0.09_O_3_ catalyst have H_2_/CO ratio greater than one which suggests that the Boudouard reaction is the predominant side reaction. CeO_2_ impregnated with yttrium increases ionic conductivity with Ce and Y bonded together, suggesting the possible presence of the CeO_2_-Y_2_O_3_ phase, which favors NiO dispersion and limits the sintering [28]. Y_2_O_3_ as a basic carrier allows Ni catalyst to be easily reduced and have a better activity [29]. Yttrium enhances smaller nickel crystallites, thereby improving the dispersion of active sites. The promotion with 0.09Y led to better activity which was linked to the formation of a solid solution of ZrO_2_-Y_2_O_3_, thereby enhancing the reduction of bulk NiO [29]. In addition, a number of oxygen vacancies and mobility can be enhanced by doping cerium with yttrium [39]. Ions of Ce^3+^ (0.97 Å) can be substituted by Y^3+^ (1.04 Å) and form a solid solution due to their similar ionic radii [39].

### 3.6. Thermogravimetric Analysis of the Used Catalyst (TGA)

**Figure 10:**
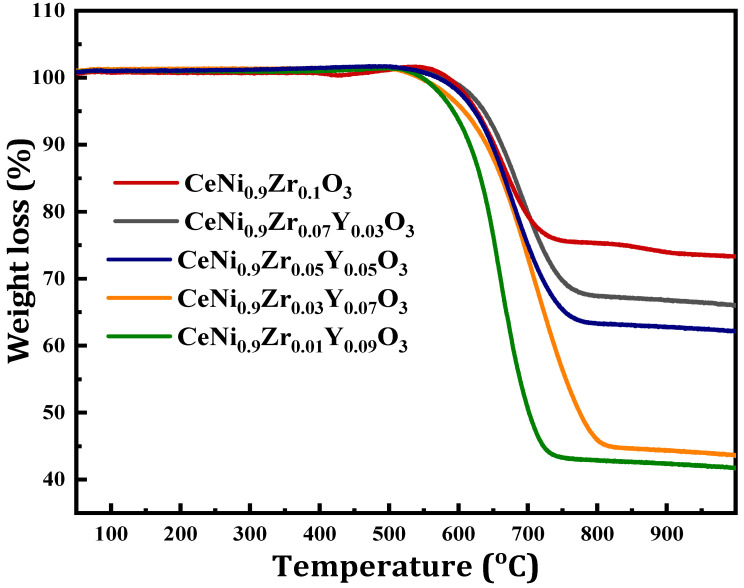
TGA Curves of perovskite catalysts CeNi_0.9_Zr_0.1−*x*_Y_*x*_O_3_ (*x* = 0, 0.03, 0.05, 0.07, and 0.09) at 800 °C.

The TGA curve of the spent catalysts was plotted in Figure 10 showing the weight loss of each catalyst expressed in percentage. The weight loss of the spent catalyst is shown to be 20%, 30%, 40%, 50%, and 55% for CeNi_0.9_Zr_0.1_O_3_, CeNi_0.9_Zr_0.07_Y_0.03_O_3_, CeNi_0.9_Zr_0.05_Y_0.05_O_3_, CeNi_0.9_Zr_0.03_Y_0.07_O_3_, and CeNi_0.9_Zr_0.01_Y_0.09_O_3_, respectively. The CeNi_0.9_Zr_0.1_O_3_ catalyst has the lowest amount of carbon compared to modified yttrium catalysts due to the former having a lesser amount of zirconium. The amount of carbon deposited on the catalyst increases with decreases in zirconium amount.

### 3.7. RAMAN Analysis

**Figure 11:**
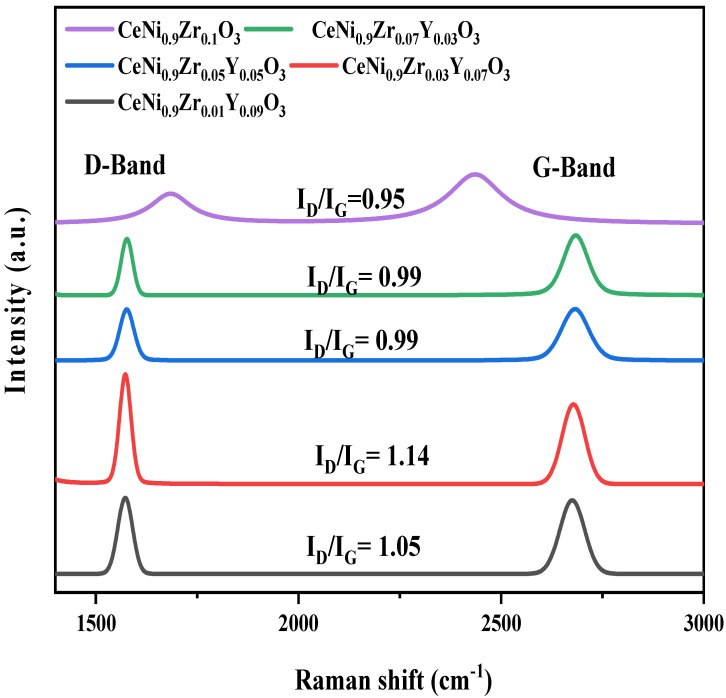
Raman Spectra of Used Perovskite Catalyst CeNi_0.9_Zr_0.1−*x*_Y_*x*_O_3_ (*x* = 0.03, 0.05, 0.07, and 0.09).

Raman spectra of the used catalysts (Figure 11) depict two bands with Raman shifts in the range of 1574 ± 5 cm^−1^ and 2650 ± 10 cm^−1^, corresponding to the D and G bands respectively. The D band is related to coke deposits with imperfect, that is, disordered structure (amorphous carbon), while the G band is related to well-ordered structure (graphitic carbon). The I_D_/I_G_ ratio is less than one since I_D_ is less than I_G_ for used catalyst CeNi_0.9_Zr_0.1−x_Y_x_O_3_ when x = 0, 0.03 and 0.05; hence, it contains imperfect (amorphous carbon) on the catalyst surface after reaction while on the other hand for CeNi_0.9_Zr_0.1−x_Y_x_O_3_ catalysts where x = 0.07 and 0.09 have more graphitic carbon as the I_D_/I_G_ ratio is greater than one for these catalysts.

## 4. Conclusions

The methane conversion activities of the best-modified catalyst (CeNi_0.9_Zr_0.01_Y_0.09_O_3_) is significantly higher than the base catalyst (CeNi_0.9_Zr_0.1_O_3_)_._ The amount of the metal ion affects activity as CeNi_0.9_Zr_0.1_O_3_ modified with 0.09Y shows an increase in activity compared to CeNi_0.9_Zr_0.1_O_3_ modified with 0.03Y. The catalyst activity increases with an increase in yttrium loading with CeNi_0.9_Zr_0.01_Y_0.09_O_3_ catalyst having the best activity with CH_4_ conversion >85% and CO_2_ conversion >90%. The H_2_/CO ratios for CeNi_0.9_Zr_0.1_O_3_ and CeNi_0.9_Zr_0.07_Y_0.03_O_3_ catalysts are less than one, which suggests that the predominant side reaction is RWGS while CeNi_0.9_Zr_0.05_Y_0.05_O_3_, CeNi_0.9_Zr_0.03_Y_0.07_O_3_, and CeNi_0.9_Zr_0.01_Y_0.09_O_3_ catalysts having H_2_/CO ratios greater than one suggests that the Boudouard reaction is the predominant side reaction. The amount of carbon deposited on the catalyst decreases with the increase in zirconium amount hence zirconium helps to improve the catalyst stability being thermally stable.

## Figures and Tables

**Table 1:** Tabular presentation of Specific surface area (SSA), Pore volume (P_v_), and Pore diameter (P_d_) of the fresh catalyst samples.

Samples	SSA (m^2^/g)	P_v_ (cm^3^/g)	P_d_ (nm)
CeNi_0.9_Zr_0.1_O_3_	9.35	0.031	12.12
CeNi_0.9_Zr_0.07_Y_0.03_O_3_	1.27	0.003	15.44
CeNi_0.9_Zr_0.05_Y_0.05_O_3_	1.62	0.003	14.43
CeNi_0.9_Zr_0.03_Y_0.07_O_3_	1.85	0.002	22.21
CeNi_0.9_Zr_0.01_Y_0.09_O_3_	2.66	0.011	29.33

**Table 2:** Summary of Nickel Particle Size Derived from TEM Analysis.

Name	Ni Particle Size
Fresh CeNi_0.9_Zr_0.1_O_3_	9.44 nm
Used CeNi_0.9_Zr_0.1_O_3_	11.68 nm
Fresh CeNi_0.9_Zr_0.01_Y_0.09_O_3_	6.66 nm
Used CeNi_0.9_Zr_0.01_Y_0.09_O_3_	7.10 nm

## Data Availability

Not applicable.
